# Artificial Intelligence in Gingival Recession Assessment and Treatment Planning: A Scoping Review

**DOI:** 10.3390/jcm15197401

**Published:** 2026-09-23

**Authors:** Paweł Sieradzki, Bartłomiej Górski

**Affiliations:** 1 Private Practice, Os.Polan 33, 61-253 Poznań, Poland; 2 Department of Periodontology and Oral Diseases, Medical University of Warsaw, 02-091 Warsaw, Poland; bartlomiej.gorski@wum.edu.pl

**Keywords:** artificial intelligence, machine learning, gingival recession, periodontal plastic surgery, periodontal imaging, clinical decision support

## Abstract

**Background/Objectives**: Artificial intelligence (AI) and machine learning (ML) are increasingly used in periodontal assessment, image analysis, outcome prediction, and clinical decision support. However, evidence specifically addressing gingival recession (GR) and related periodontal soft-tissue parameters remains fragmented and predominantly technical. This scoping review aimed to map AI/ML applications for the detection, measurement, prediction, and treatment planning of GR and to identify methodological limitations and translational evidence gaps. **Methods**: This scoping review was conducted in accordance with the JBI methodology for scoping reviews and reported according to PRISMA-ScR. Eligibility was structured according to the Population–Concept–Context framework and included English-language original studies involving human participants or human-derived clinical data and evaluating AI/ML approaches directly related to GR or to periodontal soft-tissue or anatomical parameters relevant to its assessment or management. PubMed/MEDLINE, Scopus, and Embase were utilized as the core bibliographic databases, while Google Scholar was used as an additional search source with screening restricted to the first 200 results ranked by relevance. No restrictions on publication year or start date were applied. Study characteristics, AI methods, clinical applications, validation strategies, and performance metrics were charted and descriptively synthesized. **Results**: Twelve studies were included across four main clinical domains: detection and segmentation of gingival recession and related soft-tissue features, measurement and anatomical landmark localization, prediction and risk assessment, and treatment planning or clinical decision support. AI/ML models generally demonstrated promising technical performance, including high classification and segmentation accuracy and submillimeter localization of periodontal anatomical landmarks. However, datasets, reference standards, validation strategies, and reported performance metrics were heterogeneous. Independent external validation remained limited, and most studies assessed technical performance rather than clinical utility. No study prospectively demonstrated that AI-assisted assessment or decision support improved clinician decision-making or patient outcomes. **Conclusions**: AI shows potential to support objective GR assessment, prediction, and treatment planning in gingival recession management. However, current evidence remains predominantly proof-of-concept. Multicenter external validation and prospective evaluation in real-world clinical workflows are required before routine implementation.

## 1. Introduction

Artificial intelligence (AI) and machine learning (ML) have rapidly expanded across healthcare, with applications ranging from automated image analysis and diagnostic support to risk prediction and treatment planning [1]. In dentistry, AI-based approaches have been increasingly investigated in several fields, including oral and maxillofacial radiology, orthodontics, restorative dentistry, and digital smile design [2]. Within periodontology, research has focused primarily on automated assessment of periodontal bone loss on radiographs [3] and the detection of gingival inflammation, plaque, and calculus using intraoral photographs and digital scans [4,5,6]. In contrast, the application of AI to gingival recession and related periodontal soft-tissue parameters remains comparatively underexplored.

Gingival recession (GR) is defined as the apical displacement of the gingival margin relative to the cementoenamel junction (CEJ) [7]. It is highly prevalent in adult populations and represents a common clinical finding in periodontal practice [7,8]. GR may be associated with esthetic concerns, dentin hypersensitivity, root caries, non-carious cervical lesions, and difficulties in maintaining adequate plaque control [9,10,11]. Accurate assessment of GR and associated anatomical parameters is therefore important for diagnosis, monitoring, prognosis, and treatment planning.

Clinical evaluation of gingival recession relies predominantly on measurements obtained with a periodontal probe. Although widely accepted and readily applicable, conventional probing is operator-dependent and may be affected by factors such as probe positioning, angulation, landmark identification, and examiner variability. Assessment of related parameters, including keratinized gingiva width (KGW), gingival phenotype, and the position of the gingival margin relative to the CEJ, is similarly dependent on reproducible identification of anatomical landmarks [11,12,13]. These limitations provide a rationale for developing objective and standardized approaches to periodontal soft-tissue assessment.

The increasing availability of high-resolution intraoral photography, digital intraoral scanning, and periodontal ultrasonography provides new sources of digital data that can potentially be analyzed using AI-based methods. Convolutional neural networks (CNN) vision-based deep-learning architectures, and other ML approaches have demonstrated substantial capabilities in image segmentation, anatomical landmark detection, classification, and outcome prediction across medical imaging applications [14,15,16,17,18]. In the context of periodontal soft tissues, these technologies may facilitate automated detection and measurement of GR, segmentation of keratinized gingiva, localization of periodontal landmarks, prediction of treatment outcomes, and integration of multiple clinical variables into decision-support systems.

Despite this growing technological potential, the available evidence remains fragmented and methodologically heterogeneous. Existing studies differ with respect to data sources, AI architectures, clinical objectives, reference standards, validation strategies, and reported performance metrics. Moreover, the literature spans clinically distinct applications, including photographic detection and soft-tissue segmentation, periodontal ultrasonography, risk and outcome prediction, and AI-assisted treatment planning. These applications represent different stages of the clinical pathway and therefore require separate consideration when assessing their potential clinical value.

Existing reviews of AI in dentistry and periodontology have generally addressed broader diagnostic and imaging applications. In contrast, the specific contribution of the present review is its focused mapping of AI/ML applications directly related to GR, together with periodontal soft-tissue and anatomical parameters that are directly relevant to its assessment and management. The available evidence was therefore organized into four clinical domains: (1) detection and segmentation, (2) measurement and anatomical landmark localization, (3) prediction and risk assessment, and (4) treatment planning and clinical decision support.

Therefore, the aim of this scoping review was to map and characterize the available evidence on the use of AI and ML for the detection, measurement, prediction, and treatment planning of gingival recession and related periodontal soft-tissue parameters. In addition, we aimed to identify methodological limitations, evidence gaps, and priorities for clinical translation, with particular attention to dataset representativeness, reference standards, validation strategies, potential data leakage, and demonstrated clinical utility.

## 2. Materials and Methods

### 2.1. Review Design and Reporting Framework

This scoping review was conducted in accordance with the methodological guidance for scoping reviews provided by the Joanna Briggs Institute (JBI) and is reported in accordance with the Preferred Reporting Items for Systematic Reviews and Meta-Analyses Extension for Scoping Reviews (PRISMA-ScR) [19]. The review was designed to map the extent, characteristics, and clinical applications of the available evidence on AI and ML in gingival recession assessment and management, as well as to identify methodological limitations and gaps in the existing literature. Given the heterogeneity of the available evidence in terms of study designs, input data, AI/ML approaches, clinical tasks, and reported outcomes, no quantitative synthesis or meta-analysis was planned. The review protocol was not prospectively registered, and no separate protocol was published. The conduct and reporting of the review followed the JBI methodological guidance for scoping reviews and the PRISMA-ScR reporting framework.

The eligibility criteria and review question were structured according to the Population–Concept–Context (PCC) framework. The Population comprised human participants or human-derived clinical datasets relevant to gingival recession and associated periodontal soft-tissue parameters. The Concept encompassed AI- and ML-based approaches for detection, classification, segmentation, measurement, prediction, risk assessment, treatment planning, or clinical decision support. The Context included clinical periodontology, mucogingival and periodontal plastic surgery, periodontal imaging, and other clinical settings relevant to the assessment or management of gingival recession.

### 2.2. Eligibility Criteria

Studies were considered eligible if they reported original research published in English, involved human participants or human-derived clinical data, and evaluated an AI- or ML-based approach relevant to gingival recession. Eligible applications included diagnosis, detection, classification, segmentation, measurement, prediction, prognosis, imaging, treatment planning, or clinical decision support. Studies assessing periodontal soft-tissue or anatomical parameters were considered eligible when these parameters were directly relevant to the assessment, prediction, or management of gingival recession.

For evidence-mapping purposes, a distinction was made between studies directly addressing GR as a target outcome or clinical task and studies evaluating periodontal soft-tissue or anatomical parameters with direct relevance to GR assessment or management. This distinction was maintained when interpreting the clinical applicability of the included studies.

Studies were excluded if they did not evaluate an AI/ML-based method, used conventional software without a machine-learning component, or investigated AI applications in periodontology without a direct relevance to gingival recession. Publications for which the full text could not be obtained were also excluded from the final evidence synthesis.

### 2.3. Information Sources and Search Strategy

A structured literature search was conducted in PubMed/MEDLINE, Scopus, and Google Scholar on 30 June 2026. To address the need for broader and more reproducible bibliographic coverage, the search was updated on 15 September 2026 and expanded to include Embase. No restrictions on publication year or start date were applied—all records available from database inception to the respective search dates were eligible for screening.

The search strategy was developed to capture three core concepts corresponding to the review question: (1) artificial intelligence and machine learning, (2) gingival recession and related periodontal soft-tissue conditions, and (3) diagnostic, imaging, predictive, and treatment-planning applications.

PubMed/MEDLINE, Embase and Scopus were selected to provide complementary biomedical and multidisciplinary bibliographic coverage, whereas Google Scholar was used as a supplementary source to broaden retrieval of potentially relevant records. In PubMed/MEDLINE, Medical Subject Headings (MeSH), where applicable, and free-text terms were combined using Boolean operators. The search strategy was adapted for Scopus using the TITLE-ABS-KEY field and for Embase using Emtree controlled vocabulary terms, including exploded terms where appropriate, combined with free-text terms searched in the title, abstract, and author keyword fields. Google Scholar was used as a supplementary information source using simplified combinations of terms representing the same core concepts. Given the broad scope of Google Scholar and limited reproducibility of its relevance ranking, a pragmatic approach was adopted, whereby screening was restricted to the first 200 records ranked by relevance.

The complete search strategies for all information sources, including the search terms, Boolean operators, and field tags, are provided in Supplementary Table S1. To supplement the database search and increase retrieval sensitivity, one-generation backward and forward citation tracking of all studies included after the database search was performed on 4 September 2026. Citation tracking identified 11 additional records, of which 3 met the predefined eligibility criteria and were included in the final evidence synthesis.

### 2.4. Study Selection Process

Following removal of duplicate records, study selection was performed independently by two reviewers in a two-stage process. First, titles and abstracts of all unique records were screened against the predefined eligibility criteria. Records considered potentially eligible were subsequently retrieved for full-text assessment. The full texts were independently assessed for eligibility using the same predefined criteria. Any disagreements between the reviewers at either stage were resolved through discussion and consensus. Studies that did not meet the eligibility criteria were excluded from the review.

The study selection process, including the numbers of records identified, screened, assessed for eligibility, and included in the final review, was documented using a PRISMA-ScR flow diagram.

### 2.5. Data Charting and Data Items

Data from the included studies were systematically charted to characterize the available evidence and facilitate comparison across studies. Data were charted by one reviewer (P.S.) using a structured data-charting framework and were subsequently independently checked by a second reviewer (B.G.) for completeness and accuracy. Any discrepancies identified during the verification process were resolved through discussion and consensus between the two reviewers. The extracted information included study author, year of publication, country, study design, sample and dataset characteristics, type of input data, AI/ML model or architecture, target clinical application, reference standard where reported, validation approach, reported performance metrics, main findings, and potential clinical relevance.

The extracted data were organized according to the principal clinical applications of AI/ML identified across the included studies, including detection and segmentation, measurement and anatomical landmark localization, prediction and risk assessment, and treatment planning or clinical decision support. Owing to substantial heterogeneity in study designs, datasets, AI/ML approaches, target tasks, and reported performance metrics, the findings were summarized descriptively rather than quantitatively pooled.

### 2.6. Methodological Characteristics of the Included Studies

Given the exploratory and heterogeneous nature of the evidence included in this scoping review, methodological characteristics were examined descriptively rather than summarized using an overall numerical quality score. The appraisal focused on six domains relevant to AI-based clinical research: (1) study design and population characteristics, including population representativeness; (2) dataset characteristics, including sample size, data diversity, and class balance; (3) reference standard, including the clinical validity of the reference standard and annotation procedures; (4) model development, including description of the AI/ML architecture, training strategy, and measures to limit overfitting; (5) validation strategy, including separation of training and test datasets and the use of internal or external validation; and (6) clinical applicability and reporting, including interpretability, clinical relevance of the evaluated outcomes, and assessment of potential clinical utility.

These domains were used to identify recurring methodological strengths, limitations, and evidence gaps across the included studies. Particular attention was given to external validation, dataset representativeness, annotation quality, class imbalance, participant-level independence of dataset partitions and the potential for data leakage, and evaluation of AI/ML systems against clinically meaningful outcomes. The methodological characteristics were synthesized descriptively and were not used to generate an overall quality ranking of the included studies.

### 2.7. Synthesis and Presentation of Results

Given the heterogeneity of the included studies in terms of study design, data sources, AI/ML approaches, clinical objectives, and reported performance metrics, the evidence was synthesized descriptively without quantitative pooling or meta-analysis. Studies were grouped according to their primary clinical application into four domains: (1) detection and segmentation, (2) measurement and anatomical landmark localization, (3) prediction and risk assessment, and (4) treatment planning and clinical decision support.

Study characteristics, AI/ML methods, input data, target tasks, performance metrics, validation approaches, and potential clinical applications were summarized in tabular form. Methodological characteristics and recurring evidence gaps were additionally mapped across the included studies to provide an overview of the current maturity and clinical translation of AI/ML applications relevant to gingival recession.

## 3. Results

### 3.1. Study Selection

The literature search identified 90 records: 10 from PubMed/MEDLINE, 15 from Scopus, 18 from Embase and 47 records retained from Google Scholar after screening the first 200 results ranked by relevance. Following removal of 18 duplicates, 72 unique records remained for title and abstract screening. Of these, 14 reports were considered potentially eligible and sought for full-text retrieval.

One report could not be retrieved in full text and was therefore not assessed for eligibility. The remaining 13 full-text reports were assessed against the predefined eligibility criteria, of which four were excluded. Backward and forward citation tracking of these nine studies subsequently identified 11 additional records, three of which met the eligibility criteria. Consequently, a total of 12 studies were included in the final scoping review. The complete study selection process, including the citation-tracking phase, is presented in the updated PRISMA-ScR flow diagram (Figure 1).

### 3.2. Characteristics of the Included Studies

The twelve included studies were published between 2021 and 2026, with 11 published in 2025 or 2026, and investigated heterogeneous AI/ML applications relevant to gingival recession assessment and management. The studies varied considerably in terms of study population, dataset size, input modality, AI/ML architecture, target clinical task, and validation strategy. Input data included intraoral photographs, periodontal ultrasound images and videos, intraoral scans, and structured clinical variables.

The identified applications were grouped into four principal clinical domains: (1) detection and segmentation of gingival recession and recession-related soft-tissue structures; (2) measurement and anatomical landmark localization; (3) prediction and risk assessment, including prediction of root-coverage outcomes and recession development; and (4) treatment planning and clinical decision support. The characteristics of the included studies are summarized in Table 1.

### 3.3. Detection and Segmentation

Four studies investigated AI-based detection or segmentation of gingival recession and recession-related soft-tissue features using intraoral photographs [23,27,28,29]. Two studies focused on automated segmentation and measurement of keratinized gingiva [27,28], one evaluated automated detection of gingival recession in the mandibular anterior region [23], and one assessed the classification of gingival phenotypes [29].

Chen et al. [27] analyzed 576 intraoral photographs obtained from 32 participants, including unstained images, images acquired with customized reference retainers, and images obtained after iodine staining. A DeepLabV3 model with a ResNet50 backbone was used for segmentation of keratinized gingiva. The model achieved a pixel accuracy of 93.32% and a Dice coefficient of 93.30%.

Aykol-Sahin et al. [28] evaluated deep-learning models using 1200 single-tooth images obtained from 50 patients before and after application of a disclosing agent. ResNet18, ResNet50, and MobileNetV2 architectures combined with DeepLabV3+ were assessed for identification of keratinized gingiva. ResNet50 showed the best performance, with an accuracy of 91.4%. Agreement between clinician-based measurements and the deep-learning model was high, with intraclass correlation coefficients ranging from 0.90 to 0.97 depending on jaw and gingival phenotype.

Mohamed et al. [23] evaluated automated identification of gingival recession using 700 intraoral images, equally distributed between healthy sites and sites presenting recession in the mandibular anterior region. The images were manually labeled by experts and divided into training and testing datasets. The investigated ML approaches demonstrated high classification performance, with reported accuracies ranging from 96% to 100% depending on the classifier.

Liu et al. [29] developed deep learning models to automatically classify gingival phenotype as thick, medium, or thin using standardized intraoral photographs from 1600 participants. The reference standard was established clinically using the probe transparency method. The authors compared a Vision Transformer (ViT) architecture with a conventional ResNet-18 model. The ViT model demonstrated superior diagnostic performance, achieving an area under the curve (AUC) of 1.00 for the thick phenotype, 0.98 for the thin phenotype, and 0.97 for the medium phenotype.

Overall, the available evidence indicates that photographic AI models have been investigated for three principal applications: detection of gingival recession, segmentation and measurement of keratinized gingiva, and classification of gingival phenotype. Although high technical performance was reported, the studies relied predominantly on standardized and task-specific photographic acquisition protocols, and external validation remained limited.

### 3.4. Measurement and Anatomical Landmark Localization

Four studies investigated AI-assisted analysis of periodontal ultrasound images for segmentation, anatomical landmark localization, and quantitative measurement of periodontal structures [20,21,30,31]. These applications focused on automated identification of clinically relevant landmarks, including the gingival margin (GM), cementoenamel junction (CEJ), and alveolar bone crest (ABC) as well as quantitative assessment of soft- and hard-tissue dimensions.

Qi et al. [20] evaluated a U-Net convolutional neural network using 1580 periodontal ultrasound images obtained from 184 teeth in 29 participants. The model was trained to identify periodontal structures and landmarks from which three quantitative indices were derived: gingival recession (iGR), gingival height (iGH), and alveolar bone level (iABL). On the internal test set, mean absolute landmark localization errors were below 70 μm, and more than 96% of derived measurements were within 200 μm of the reference annotations. Model performance remained below a 1 mm error threshold when evaluated on an additional dataset, while limited cross-device testing suggested some degree of generalizability.

Piao et al. [21] developed a deep-learning architecture for simultaneous segmentation of gingiva, enamel, cementum, and alveolar bone and localization of the GM, CEJ, and ABC. The study included 752 ultrasound images in the internal dataset and an external test set of 111 images acquired using a different ultrasound device and imaging parameters. Median Dice similarity coefficients were 0.891 for internal testing and 0.841 for external testing. Landmark localization errors were 0.06 mm for the CEJ, 0.08 mm for the ABC, and 0.06 mm for the GM. The model showed significantly lower localization errors than both junior and senior dentists, with high agreement between AI-derived and reference measurements.

Nguyen et al. [31] evaluated a deep learning approach to automatically localize the CEJ using 1400 periodontal ultrasound images obtained from 36 participants. Among the evaluated architectures, a customized DenseNet201 model demonstrated the best performance. To establish a robust reference standard, annotations from five clinical raters were synthesized using the STAPLE algorithm. The model achieved a mean localization difference of 0.26 mm compared with the STAPLE estimate, performing well within the range of inter-rater variability. Furthermore, AI-derived measurements of the CEJ-ABC distance correlated strongly with manual clinical labeling (R = 0.933), demonstrating 95% limits of agreement between −0.68 and 0.57 mm.

Kumaralingam et al. [30] extended AI-assisted periodontal ultrasonography from static-image analysis to continuous video assessment. The authors developed OralSAM, a foundation model for automated segmentation and quantitative assessment of the periodontium in intraoral ultrasound videos. The dataset comprised 158 videos, each containing approximately 500 frames, obtained from 30 orthodontic patients. The model simultaneously segmented gingiva, enamel, alveolar bone, and cementum, enabling automated extraction of alveolar bone level (ABL), gingival thickness (GT), and alveolar bone thickness (ABT). Agreement with expert annotations was high, with intraclass correlation coefficients of 0.893 for ABL, 0.918 for GT, and 0.848 for ABT. The system achieved a processing speed of approximately 13 frames per second.

Collectively, these studies demonstrate a progression of AI-assisted periodontal ultrasonography from landmark localization and static multi-tissue segmentation toward dynamic, video-based quantitative assessment of periodontal structures. High technical performance and submillimeter localization accuracy were reported across several tasks. However, most evaluations remained based on internal datasets or limited cross-device testing, and broad independent external validation remains lacking.

### 3.5. Prediction and Risk Assessment

Three studies investigated AI/ML approaches for prediction or risk assessment in clinically distinct settings [22,24,26]. These applications included prediction of root-coverage outcomes following mucogingival surgery, estimation of gingival recession risk associated with orthodontic treatment, and patient-specific simulation of long-term recession-related periodontal changes.

Sun et al. [26] developed a YOLO-v8-based model to predict maximum root coverage from preoperative intraoral photographs. The dataset comprised 408 photographs representing 963 teeth and was divided into training, validation, and test sets at a 7:2:1 ratio. The model achieved a Dice coefficient of 98.27% and an intersection over union of 96.58% for segmentation. Predicted maximum root coverage was additionally compared with the actual gingival margin position observed on postoperative photographs at 6 months, with a mean error of 0.91 ± 1.32 pixels.

Anishya et al. [22] evaluated a neural-network model for predicting gingival recession in 65 patients who had undergone camouflage orthodontic treatment. The model incorporated orthodontic and anatomical variables related to the mandibular anterior region and was trained using an 80:20 training-to-testing split. Although an overall accuracy of 92% was reported, the true-positive rate was 50%, with a corresponding false-positive rate of 50%, indicating substantially lower performance for identification of recession-positive cases than suggested by overall accuracy alone.

Gunpinar et al. [24] analyzed clinical, demographic, behavioral, and intraoral-scan data from 1057 patients. K-means clustering was used to stratify patients into low-, medium-, and high-risk periodontal groups, while gingival curves were extracted from intraoral scans using Dijkstra’s algorithm. The resulting system generated patient-specific three-dimensional simulations of gingival recession progression over time horizons ranging from 1 to 20 years. Twenty-five periodontal specialists evaluated the clinical similarity of the generated models, with 36–44% reporting that they were “satisfied” and 12–20% “very satisfied”. A quantitative measure of predictive accuracy for long-term recession progression was not reported.

Overall, predictive applications encompassed markedly different clinical objectives and outcome definitions, ranging from postoperative anatomical outcomes to risk classification and long-term visualization. This heterogeneity precluded direct comparison of model performance across studies.

### 3.6. Treatment Planning and Clinical Decision Support

One study investigated the use of ML for treatment planning and clinical decision support in gingival recession surgery [25]. Karagöz et al. [25] developed a classification model to support selection among four mucogingival surgical approaches: gingival unit graft (GUG), coronally advanced flap (CAF), CAF combined with a connective tissue graft (CAF+CTG), and CAF combined with platelet-rich fibrin (CAF+PRF).

The analysis included 132 teeth with gingival recession. To predict the type of surgery, the model incorporated preoperative baseline variables: baseline recession depth and width, preoperative width of keratinized gingiva, plaque index, initial buccogingival tissue thickness, frenulum position, tooth mobility, and recession type. Three ML approaches—logistic regression, multilayer perceptron, and random forest—were evaluated. Random forest demonstrated the highest overall performance, with an accuracy of 86.4%, and was subsequently used as the basis for the decision-support tool. Performance varied across treatment categories, with the highest category-specific accuracy reaching 97.8% for CAF+PRF.

This study represents an application of ML beyond automated detection or measurement, integrating multiple clinical variables to support selection of a surgical approach. However, the model was evaluated within the dataset used for its development, and the study did not assess whether use of the decision-support tool influenced clinician decision-making or treatment outcomes in clinical practice.

### 3.7. Methodological Characteristics and Evidence Gaps

Methodological characteristics varied considerably across the included studies. Although most studies provided sufficient descriptions of the AI/ML architecture and model-development procedures, recurring limitations were identified in dataset representativeness, validation strategies, reference-standard development, reporting of class balance, and assessment of clinical utility.

Although some recent studies included comparatively large cohorts, including 1600 participants in Liu et al. [29] and 1057 participants in Gunpinar et al. [24], several studies relied on relatively small or single-center datasets [22,23,25,27,28]. External or cross-device validation remained uncommon, and most studies relied predominantly on internal test datasets. Piao et al. [21] evaluated performance using images acquired with a different ultrasound device and acquisition parameters, while Qi et al. [20] additionally assessed model performance on an outside dataset.

Reporting of patient-level independence between training, validation, and test partitions was also limited. This issue is particularly relevant in studies in which multiple images, teeth, or imaging frames originated from the same participant. Unless observations from the same individual are kept within a single dataset partition, information leakage may occur and model performance may be overestimated. Kumaralingam et al. [30] explicitly reported subject-level separation across video partitions; however, participant-level independence was not consistently described across the remaining studies.

The quality and reporting of reference standards also varied. Some studies relied on annotations provided by a single examiner without reporting inter-rater reliability [23,26], whereas other studies implemented more rigorous ground-truth establishment. Nguyen et al. [31], for example, synthesized annotations from five clinical raters using the STAPLE algorithm, while other ultrasound-based studies also incorporated expert annotation or review procedures [20,21]. Reporting of class distribution and strategies to address class imbalance was inconsistent, particularly in studies based on smaller datasets [22,23].

Clinical validation remained limited. A few studies extended technical model evaluation toward clinically relevant assessment: Sun et al. [26] compared predicted root coverage with the postoperative gingival margin observed at 6 months, whereas Gunpinar et al. [24] obtained specialist assessments of the similarity of patient-specific recession simulations to clinical presentations. Despite advances in technical capability, including near-real-time ultrasound-video processing [30], none of the included studies prospectively evaluated whether integration of an AI/ML system into a clinical workflow improved clinician decision-making or patient outcomes. Overall, the evidence map indicates that current AI/ML applications relevant to gingival recession are characterized predominantly by technical development and internal performance evaluation, with comparatively limited evidence regarding external generalizability and real-world clinical utility (Table 2).

## 4. Discussion

### 4.1. Principal Findings and Evidence Landscape

This scoping review mapped the available evidence on AI and ML applications relevant to the assessment and management of gingival recession and associated periodontal soft-tissue parameters. Nine studies were identified, encompassing four principal clinical domains: detection and segmentation, measurement and anatomical landmark localization, prediction and risk assessment, and treatment planning or clinical decision support. The evidence base is recent and heterogeneous, with seven of the twelve included studies published in 2025 or 2026 and substantial variation in data modalities, AI/ML architectures, target tasks, and validation strategies.

Across these domains, AI/ML systems generally demonstrated promising technical performance. Image-based models achieved high accuracy in gingival recession detection and keratinized gingiva segmentation, and gingival phenotype classification [23,27,28], while ultrasound-based approaches enabled automated segmentation and submillimeter localization, quantitative periodontal measurements, and video-based assessment [20,21]. Predictive applications extended beyond diagnostic assessment to estimation of root-coverage outcomes, orthodontic recession risk, and visualization of recession-related progression [22,24,26]. In addition, one study explored ML-based support for selecting among mucogingival surgical approaches [25]. Collectively, these studies illustrate an emerging transition from automated recognition and measurement toward prediction and clinical decision support.

However, the apparent technical performance of these models should not be equated with demonstrated clinical effectiveness. Although some recent studies included larger datasets or more advanced imaging approaches, most applications were still evaluated within single-center or internally derived datasets, and independent external validation remained limited. Reference-standard and annotation procedures were also inconsistently reported. Moreover, none of the included studies evaluated whether implementation of an AI/ML system within a prospective clinical workflow improved clinician decision-making or patient outcomes. Thus, the current literature primarily demonstrates technical feasibility rather than readiness for routine clinical implementation.

The principal finding of this scoping review is therefore not that AI has already established a clinical role in gingival recession management, but that several potentially relevant applications have emerged across different stages of the clinical pathway while important translational gaps remain. The most immediate research priority is to move from internally validated proof-of-concept models toward externally validated, clinically tested systems evaluated in representative patient populations and real-world periodontal workflows.

### 4.2. Clinical Application of AI/ML in Gingival Recession

AI-based analysis of intraoral photographs represents the most readily accessible application identified in this review, as it can potentially build on imaging already incorporated into routine dental documentation. Four studies addressing photographic analysis were identified and covered three related but distinct tasks: segmentation and measurement of keratinized gingiva [27,28], detection of gingival recession [23], and classification of gingival phenotype [29]. Although all three studies reported high technical performance, their potential clinical roles should be considered separately.

For keratinized gingiva assessment, Chen et al. [27] and Aykol-Sahin et al. [28] demonstrated that deep-learning models can identify the keratinized gingival region on standardized intraoral photographs with high segmentation accuracy. Importantly, these studies extended beyond image classification by comparing AI-derived measurements with clinically or photographically determined reference measurements. Such approaches may offer advantages in terms of standardization, reproducibility, and longitudinal documentation, particularly when repeated soft-tissue measurements are required. However, their performance was established under controlled imaging conditions, including calibration or staining procedures, and therefore cannot yet be assumed to translate directly to routine photographs acquired with different cameras, lighting conditions, magnifications, or operators.

Automated detection of gingival recession represents a different clinical application. Z et al. [23] reported high classification performance in distinguishing healthy sites from recession-affected mandibular anterior teeth. A photograph-based screening approach could potentially facilitate identification of recession in settings where comprehensive periodontal examination is not routinely performed. Nevertheless, the study addressed a relatively restricted binary classification task using a balanced dataset of 700 images from a single source. Consequently, the reported performance should not be interpreted as evidence that the model can reliably identify the broader clinical spectrum of gingival recession across different tooth types, recession severities, anatomical conditions, and image-acquisition environments.

Liu et al. [29] extended photographic AI analysis to automated classification of gingival phenotype using standardized intraoral photographs from 1600 participants. The large cohort and high reported AUC values suggest that deep-learning models may be capable of identifying clinically relevant soft-tissue phenotypes from photographic data. However, gingival phenotype classification should be distinguished from direct detection or prediction of gingival recession. Although phenotype represents an anatomical parameter relevant to recession assessment and treatment planning, the study did not demonstrate that AI-based phenotype classification improved prediction of recession development or clinical outcomes.

Taken together, these studies establish the technical feasibility of photographic AI for automated assessment of recession-related soft-tissue features but provide limited evidence of generalizability. The available applications now extend beyond binary recession detection and keratinized gingiva segmentation to phenotype classification; nevertheless, they remain largely dependent on standardized, task-specific photographic protocols. Future studies should therefore prioritize standardized image acquisition and annotation protocols, external validation across different clinical centers and imaging devices, and direct comparison with conventional clinical measurements. Ultimately, the relevant question is not only whether an algorithm can accurately segment or classify an image, but whether automated photographic assessment provides reproducible and clinically meaningful information under real-world conditions.

#### 4.2.1. AI-Assisted Periodontal Ultrasonography

Among the applications identified in this review, AI-assisted periodontal ultrasonography appears particularly promising for objective and quantitative assessment of periodontal anatomy. Unlike conventional two-dimensional photography, ultrasound imaging enables visualization of both soft- and hard-tissue structures, including the gingival margin, cementoenamel junction, and alveolar bone crest. Four studies investigated this field, demonstrating a progression from anatomical landmark localization and static image segmentation toward automated quantitative assessment of continuous ultrasound video sequences [20,21,30,31]. Qi et al. [20] and Piao et al. [21] demonstrated that deep-learning models can simultaneously segment periodontal structures, localize clinically relevant anatomical landmarks, and derive quantitative measurements relevant to gingival recession assessment. The precision reported in these studies is noteworthy, with submillimeter landmark localization and, in Piao et al. [21], lower localization errors than those observed for junior and senior dentists. Both studies also provided some evidence beyond conventional internal testing, although such cross-device or outside-dataset evaluation should not be considered equivalent to broad multicenter external validation.

Nguyen et al. [31] provided additional evidence for automated CEJ localization in periodontal ultrasound images. An important methodological strength of this study was the establishment of the reference standard using annotations from five clinical raters synthesized with the STAPLE algorithm. The model achieved a mean localization difference of 0.26 mm relative to the consensus estimate, and AI-derived CEJ–ABC measurements correlated strongly with manual clinical labeling. This study illustrates the importance of robust ground-truth establishment in AI models designed for anatomical landmark identification.

More recently, Kumaralingam et al. [30] extended this concept from static image analysis to continuous ultrasound video. Their OralSAM model enabled automated segmentation of multiple periodontal structures and extraction of alveolar bone level, gingival thickness, and alveolar bone thickness across consecutive frames, with high agreement with expert measurements and a processing speed of approximately 13 frames per second. This represents a potentially important step toward dynamic and near-real-time periodontal imaging.

This potential is clinically relevant because the assessment of gingival recession is inherently linked to the identification of anatomical reference points. Automated localization of the gingival margin and cementoenamel junction may facilitate objective recession measurements, while simultaneous identification of the alveolar bone crest and assessment of gingival and alveolar bone thickness could provide additional information on the relationship between soft-tissue position and underlying periodontal anatomy. Thus, AI-assisted ultrasonography may ultimately provide a more comprehensive anatomical assessment than methods based exclusively on surface visualization.

Nevertheless, the evidence remains preliminary. Despite the expansion from two to four eligible ultrasound studies, datasets remain relatively limited and heterogeneous in terms of devices, acquisition protocols, populations, and model architectures. Most evaluations were based predominantly on internal datasets, with only limited cross-device or outside-dataset testing. Moreover, the studies primarily evaluated segmentation and landmark-localization accuracy rather than whether AI-assisted ultrasound measurements improve diagnosis, treatment planning, longitudinal monitoring, or patient outcomes.

Future research should therefore evaluate these systems prospectively across multiple centers, operators, ultrasound devices, and patient populations, with standardized reference annotations and direct comparison with established clinical measurements. Attention should be given to reproducibility across devices and operators and to determining whether the additional anatomical information provided by AI-assisted ultrasonography translates into clinically meaningful changes in periodontal decision-making.

#### 4.2.2. Predictive Models and Risk Assessment

Predictive applications represent a potentially important extension of AI beyond automated detection and measurement, but the three studies identified in this review addressed substantially different clinical questions and used different outcome definitions [22,24,26]. These included prediction of maximum root coverage following mucogingival surgery, estimation of gingival recession risk associated with orthodontic treatment, and patient-specific visualization of recession-related changes over extended time horizons. Consequently, their reported performance metrics are not directly comparable and should be interpreted within the context of the specific clinical task.

Among these applications, the model developed by Sun et al. [26] was the most directly linked to a clinically observed postoperative outcome. The YOLO-v8-based approach used preoperative intraoral photographs to estimate maximum root coverage and compared model predictions with the gingival margin observed 6 months after surgery. This application is clinically relevant because reliable preoperative estimation of achievable root coverage could support prognosis, treatment planning, and communication of realistic treatment expectations. However, both model development and postoperative evaluation were performed using data from the same center and standardized imaging conditions. The reported agreement therefore demonstrates promising internal performance but does not establish generalizability across different patient populations, surgical protocols, operators, or photographic conditions.

Prediction of recession risk in orthodontic patients represents a different challenge. Anishya et al. [22] reported an overall accuracy of 92%; however, the true-positive rate was only 50%. This discrepancy illustrates the limitations of relying on overall accuracy when evaluating models intended to identify clinically relevant but potentially less frequent outcomes. In such settings, sensitivity, specificity, predictive values, discrimination measures, and calibration are necessary to determine whether a model provides clinically useful risk estimates. The findings therefore support the feasibility of incorporating periodontal and orthodontic variables into ML-based risk assessment, but do not yet establish sufficiently reliable prediction of recession at the individual-patient level.

Gunpinar et al. [24] explored a broader concept by combining periodontal risk stratification with patient-specific three-dimensional simulations of recession-related changes over periods ranging from 1 to 20 years. Such visualization may have potential value for communicating periodontal risk and illustrating possible future changes to patients. However, the generated long-term simulations were not validated against longitudinal clinical observations over the corresponding time horizons. Assessment by periodontal specialists therefore provides information on the perceived clinical plausibility or realism of the simulations rather than evidence of long-term predictive accuracy. This distinction is important when considering the potential role of generative or simulation-based approaches in clinical communication and decision support.

Taken together, these studies indicate that AI-based prediction in gingival recession is moving toward clinically relevant questions, but remains at an early stage of validation. Future models should be evaluated against prospectively collected clinical outcomes, with adequate reporting of discrimination and calibration, external validation in independent populations, and assessment of whether predicted risks or outcomes meaningfully influence clinical decisions. For surgical applications, models should ultimately be evaluated not only for their ability to reproduce postoperative anatomical outcomes, but also for their capacity to provide individualized and clinically interpretable estimates that can support shared decision-making.

#### 4.2.3. AI-Assisted Treatment Planning and Decision Support

The application of AI to treatment planning represents a further step toward clinically integrated decision support. Karagöz et al. [25] investigated whether multiple recession-related clinical and anatomical variables could be integrated using ML to support selection among four mucogingival surgical approaches. This approach illustrates the potential of ML to integrate multiple clinical parameters that must ordinarily be considered simultaneously when selecting a surgical strategy for gingival recession.

The potential value of such systems lies not in replacing clinician judgment, but in providing structured and reproducible support for complex therapeutic decisions. Selection of a root-coverage procedure depends on the interaction of multiple patient-, tooth-, and site-related factors, and the relative importance assigned to these variables may differ among clinicians. An appropriately validated decision-support model could potentially assist in standardizing this process, particularly in complex cases or as an educational tool. However, classification of a treatment choice should not be considered equivalent to prediction of treatment success. A model may reproduce the procedure selected within its development dataset without demonstrating that the recommended procedure produces the best clinical outcome for an individual patient.

This distinction is particularly important in interpreting the current evidence. The model developed by Karagöz et al. [25] was evaluated within the available dataset, and no prospective study assessed whether AI-assisted recommendations improved treatment selection, root-coverage outcomes, esthetic results, patient-reported outcomes, or decision consistency compared with conventional clinician-based planning. Furthermore, the appropriateness of an AI recommendation ultimately depends on the validity of the treatment labels used for model training. If these labels primarily reflect previous clinician choices rather than outcome-based evidence, the model may learn existing decision patterns without establishing their clinical superiority.

Future decision-support systems should therefore be trained and validated using well-defined clinical criteria and, ideally, treatment outcomes rather than treatment selection alone. Prospective studies comparing AI-assisted and conventional planning, together with external validation across clinicians and centers, will be required to determine whether these systems provide incremental clinical value. Explainability will also be particularly important in this setting, as clinicians should be able to understand which variables contributed to a recommendation before incorporating it into patient-specific treatment planning.

### 4.3. Methodological Gaps and Barriers to Clinical Translation

Despite encouraging technical performance across several applications, the evidence mapped in this review reveals recurring methodological limitations that currently restrict the clinical translation of AI/ML systems for gingival recession. The most important gaps concern dataset representativeness, external validation, reference-standard development, reporting of model performance, and evaluation of clinical utility. These limitations are particularly relevant because performance achieved under controlled development conditions may not be maintained when models are applied to more heterogeneous patient populations, imaging systems, operators, and clinical settings.

Dataset size and representativeness remain major concerns. Although some studies included comparatively large cohorts, including 1600 participants in Liu et al. [29] and 1057 participants in Gunpinar et al. [24], several studies were based on relatively small patient populations or single-center datasets [22,23,25,27,28]. This distinction is important because multiple observations derived from the same patient do not provide the same degree of biological and clinical diversity as observations obtained from independent individuals. Moreover, limited variation in patient characteristics, anatomical presentations, imaging devices, and acquisition protocols may allow models to perform well within a development dataset while reducing their generalizability to external populations. Future studies should therefore prioritize multicenter datasets representing a broader spectrum of recession phenotypes, tooth types, patient characteristics, and imaging conditions.

A related methodological concern is the independence of observations used for model development and evaluation. In datasets containing multiple teeth, photographs, or imaging frames from the same participant, splitting observations rather than participants across training and test datasets may result in information leakage. Because patient-level partitioning was not consistently reported across the included studies, the extent to which this potential source of bias was avoided could not always be determined. Kumaralingam et al. [30] explicitly reported subject-level separation across video partitions, illustrating an approach that should be consistently adopted and transparently reported in future studies.

External validation was another major limitation. Most models were evaluated using internal train-validation-test partitions derived from the same underlying dataset. Some ultrasound-based studies included cross-device or outside-dataset testing [20,21], but independent external validation across different patient populations and clinical centers remained uncommon. External validation should involve genuinely independent data that reflect differences in patient populations, clinical centers, operators, devices, and acquisition protocols. The definition and quality of the reference standard also require greater attention. AI models can reproduce only the labels and measurements on which they are trained, making annotation quality particularly important for tasks involving anatomical boundaries or clinical classification. Across the included studies, reference standards ranged from single-examiner annotations to more structured expert-review procedures. Nguyen et al. [31] represents a notable example of a more rigorous approach, using the STAPLE algorithm to integrate annotations from five clinical raters into a consensus reference standard. Future studies should clearly describe annotator expertise, annotation procedures, disagreement resolution, and reproducibility of the reference standard.

Model evaluation and reporting were similarly heterogeneous. Overall accuracy was frequently emphasized, although this metric may provide an incomplete representation of performance, particularly when outcome classes are imbalanced. The included studies used heterogeneous performance metrics, including accuracy, Dice similarity coefficients, IoU, AUC, ICC, and landmark localization errors. These metrics assess fundamentally different model tasks and cannot be directly compared across studies. Reporting should therefore be aligned with the intended clinical use of the model rather than relying on a single global performance metric. Depending on the clinical task, sensitivity, specificity, positive and negative predictive values, discrimination, calibration, Dice coefficients, intersection-over-union measures, and clinically interpretable measurement errors may be more informative. Reporting should therefore be aligned with the intended clinical use of the model rather than relying on a single global performance metric.

Most importantly, technical validation should be distinguished from clinical validation. None of the included studies prospectively evaluated whether integration of an AI/ML system into a clinical workflow improved diagnostic consistency, treatment selection, treatment outcomes, patient-reported outcomes, or efficiency of care. Even technically advanced systems capable of rapid or near-real-time processing [30] should not be considered clinically validated in the absence of evidence demonstrating incremental benefit in actual patient care.

Additional barriers to clinical translation include model interpretability, integration with existing digital workflows, interoperability between imaging systems, and the need for transparent reporting. These considerations become increasingly important as AI applications progress from automated measurement toward risk prediction and treatment recommendations, where the consequences of model errors may be more clinically significant. Taken together, the evidence indicates that the next phase of research should focus less on demonstrating increasingly high performance within individual datasets and more on establishing reproducibility, generalizability, clinical validity, and incremental value under real-world conditions.

### 4.4. Future Research Directions

The evidence mapped in this scoping review suggests that future research should progress from proof-of-concept model development toward clinically oriented validation and implementation. The main priority is the development of larger, multicenter datasets that capture the heterogeneity encountered in periodontal practice, including different recession phenotypes, tooth types, patient characteristics, operators, imaging devices, and acquisition protocols. Such datasets should be accompanied by clearly defined reference standards and, where appropriate, annotations performed by multiple calibrated experts.

External validation should become a routine component of model evaluation. Models developed at one center should be tested on genuinely independent populations and, for image-based applications, across different cameras, scanners, ultrasound devices, and acquisition settings. Prospective evaluation is subsequently required to determine whether performance observed in retrospective datasets is maintained when AI systems are incorporated into routine clinical workflows.

A further direction is the integration of multimodal data. Current studies generally rely on a single data modality, such as intraoral photographs, ultrasound images, digital scans, or structured clinical variables. Combining imaging with periodontal measurements, anatomical characteristics, patient-level risk factors, and treatment-related variables may enable more individualized models for recession risk assessment, prognosis, and treatment planning. Longitudinal datasets will be particularly important for distinguishing models that simulate plausible future changes from those that can reliably predict actual disease progression or treatment outcomes.

For predictive and decision-support applications, future studies should prioritize clinically meaningful endpoints and transparent model interpretation. Models intended to estimate root coverage or recession risk should report appropriate measures of discrimination and calibration and should be validated against prospectively observed outcomes. Similarly, systems intended to support surgical treatment selection should be evaluated according to treatment outcomes rather than their ability to reproduce previous clinician choices alone. Explainable approaches may further facilitate clinical adoption by allowing clinicians to understand which variables contribute to individual predictions or recommendations. For clinical decision-support applications, explainable AI (XAI) may be particularly important because clinicians should be able to assess whether a model output is clinically plausible and consistent with established clinical principles. Future studies should therefore evaluate both inherently interpretable models and post hoc explanation methods and determine whether these approaches improve clinician understanding, trust, and decision quality.

Ultimately, the clinical value of AI should be assessed through prospective comparative studies of AI-assisted versus conventional care. Relevant outcomes may include diagnostic and measurement reproducibility, treatment-planning consistency, prediction accuracy, clinician efficiency, patient communication, treatment outcomes, and patient-reported outcomes. Such studies would allow the field to move beyond algorithm performance and determine whether AI provides measurable incremental value in the management of gingival recession.

### 4.5. Strengths and Limitations of the Scoping Review

This scoping review has several strengths. It provides a structured mapping of the emerging evidence on AI/ML applications specifically relevant to gingival recession and associated periodontal soft-tissue parameters, encompassing diagnostic, measurement, predictive, and treatment-planning applications. The review included multiple data modalities, including intraoral photographs, periodontal ultrasound images and videos, digital intraoral scans, and structured clinical variables, thereby providing a broad overview of the technological approaches currently being explored. In addition, study selection was performed independently by two reviewers, and methodological characteristics were mapped across clinically relevant domains without reducing heterogeneous studies to a single numerical quality score. Backward and forward citation tracking was additionally performed to increase the sensitivity of literature identification and resulted in the inclusion of three additional eligible studies.

Several limitations should also be acknowledged. First, the evidence base was small, with only twelve studies meeting the eligibility criteria, and substantial heterogeneity in study designs, AI/ML architectures, input data, target tasks, and reported performance metrics precluded meaningful quantitative synthesis. However, identifying such heterogeneity and gaps in the available literature is consistent with the mapping purpose of a scoping review. Second, the search was restricted to publications in English, which may have resulted in language bias and the omission of relevant studies published in other languages.

Third, although PubMed/MEDLINE, Scopus and Embase were systematically searched, Web of Science was not included as an information source. Google Scholar was used as a supplementary source, but screening was restricted to the first 200 results ranked by relevance. Given the dynamic ranking and broad coverage of Google Scholar, potentially eligible records appearing beyond this threshold or indexed exclusively in databases not included in our search may therefore have been missed. In addition, one potentially relevant report could not be retrieved in full text and could not be assessed for eligibility. Furthermore, three eligible studies were identified only through backward and forward citation tracking. Although citation tracking increased the sensitivity of the review, this finding also indicates that the original database search may not have captured the full breadth of literature addressing periodontal soft-tissue and anatomical parameters indirectly relevant to gingival recession.

Finally, no formal validated risk-of-bias instrument was applied. Instead, methodological characteristics relevant to AI-based clinical research were descriptively mapped across six domains to identify recurring strengths, limitations, and evidence gaps. This approach is consistent with the primary objective of the present review, which was to characterize the scope and nature of the available evidence rather than to estimate a pooled treatment effect or formally grade certainty of evidence. Nevertheless, the absence of a formal risk-of-bias assessment should be considered when interpreting the methodological observations presented in this review.

## 5. Conclusions

This scoping review mapped the emerging applications of artificial intelligence and machine learning in the assessment and management of gingival recession and associated periodontal soft-tissue parameters. The available evidence spans four principal domains: detection and segmentation, measurement and anatomical landmark localization, prediction and risk assessment, and treatment planning or clinical decision support. Across these applications, AI/ML models demonstrated promising technical performance, particularly in image-based soft-tissue assessment and AI-assisted periodontal ultrasonography.

However, the current evidence base remains limited and predominantly reflects technical feasibility rather than established clinical utility. Most studies were based on single-center or internally derived datasets, independent external validation remained limited, and reference standards and performance metrics were heterogeneous. The absence of prospective evaluations within real-world clinical workflows restricts conclusions regarding generalizability and clinical effectiveness. Future research should prioritize larger multicenter datasets, rigorous participant-level data separation, standardized reference standards, independent external validation, and longitudinal assessment of clinically meaningful outcomes. Prospective comparisons of AI-assisted and conventional care are required to determine whether these systems improve measurement reproducibility, risk prediction, treatment-planning consistency, clinical decision-making, or patient outcomes. Such evidence is required to determine whether AI can provide measurable incremental value in the diagnosis, prognosis, treatment planning, and monitoring of gingival recession (Figure 2).

## Figures and Tables

**Figure 1:**
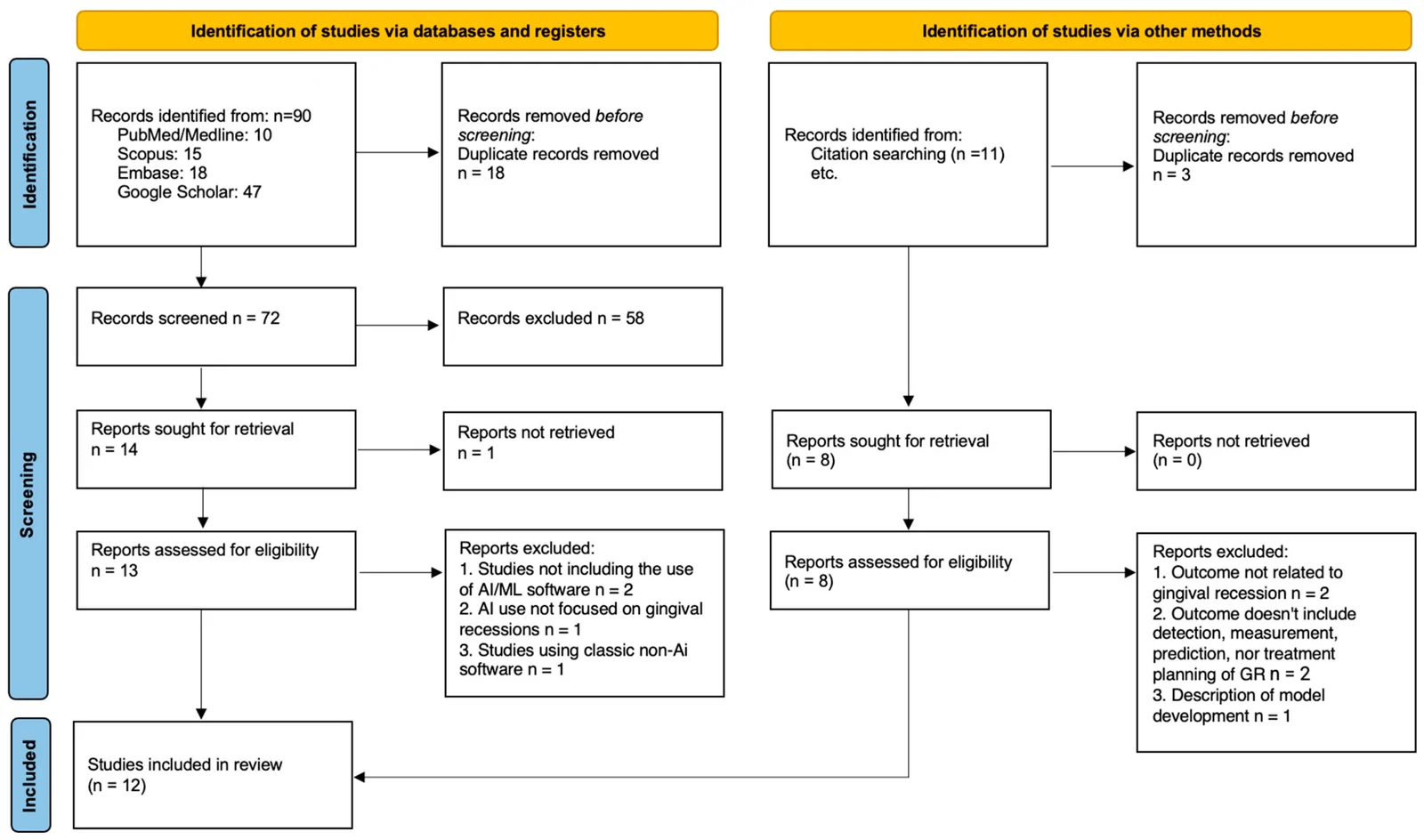
PRISMA-ScR flow diagram illustrating the study identification, screening, eligibility assessment, and inclusion process.

**Figure 2:**
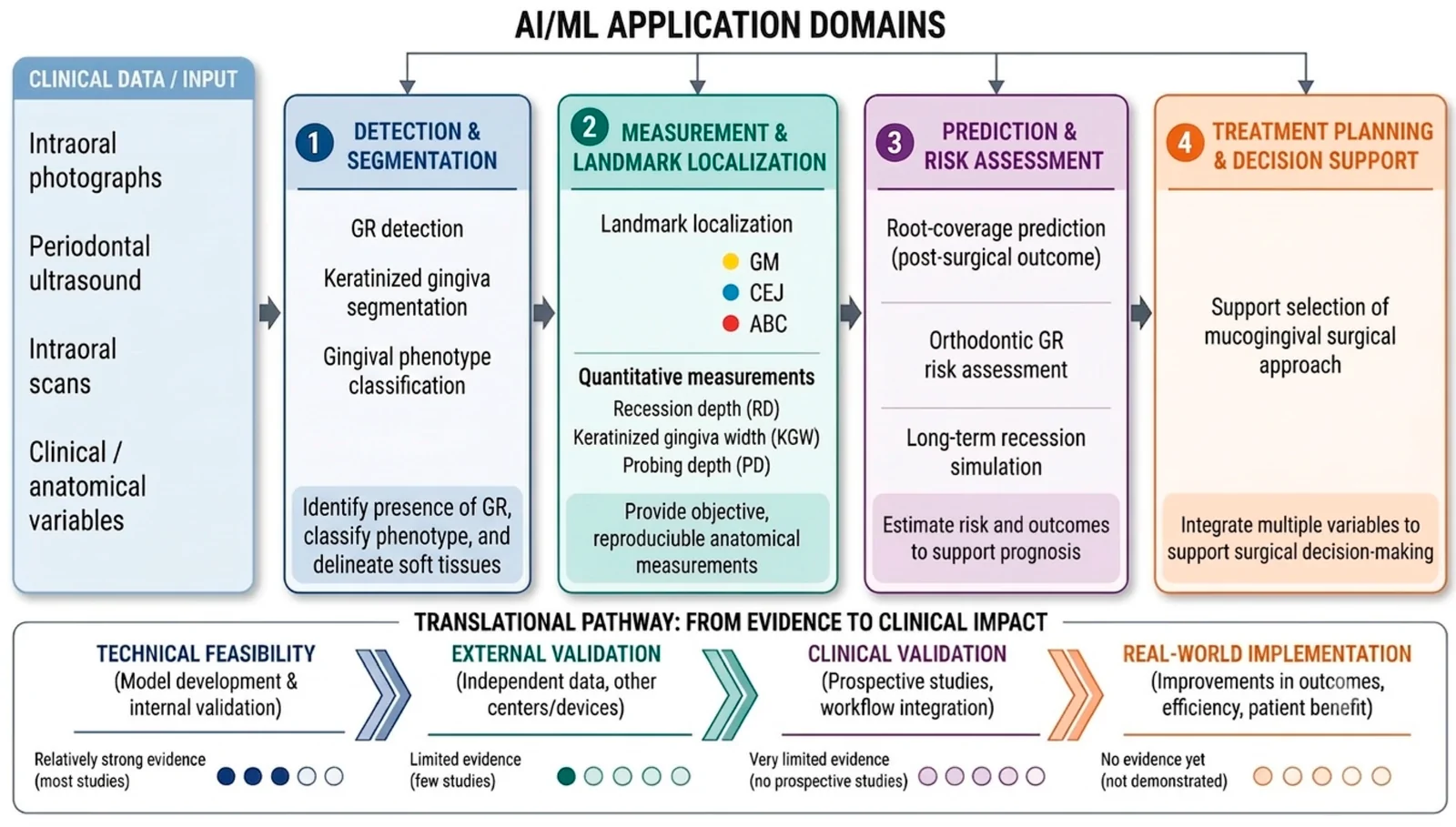
AI applications and translational pathway in GR management. The upper panel illustrates the four principal clinical domains of AI/ML applications identified in the review. The lower panel presents the translational pathway from technical feasibility to real-world clinical impact. The colored arrows and corresponding dot scales indicate the current level of evidence supporting each stage, ranging from relatively strong evidence for technical model development (blue) to a lack of evidence for real-world implementation (orange).

**Table 1:** Characteristics and main findings of the included studies. The included studies were mapped according to their clinical domain, AI/ML approach, dataset and input modality, target task, principal performance findings, and potential clinical application.

Study	Country/Year	Clinical Domain	AI/ML Approach	Dataset/Input (Npatients/Nteeth/Nimages)	Target Task	Main Performance/Findings	Potential Clinical Application
Qi et al. [20]	USA/2025	Measurement and landmark localization	U-Net CNN	1580 periodontal ultrasound frames from 184 teeth	Detection of GM, CEJ, and ABC; calculation of iGR, iGH, and iABL	Mean landmark localization errors < 70 μm; >96% of derived measurements within 200 μm of reference annotations	Automated periodontal ultrasound interpretation and quantitative periodontal measurements
Piao et al. [21]	South Korea/2026	Measurement and landmark localization	Dual-stream CNN; comparison with U-Net, U-Net++, DeepLabV3, and MA-Net	752 internal + 111 external periodontal ultrasound images	Segmentation of gingiva, enamel, cementum, and alveolar bone; localization of GM, CEJ, and ABC	DSC 0.891 internal and 0.841 external; localization error: CEJ 0.06 mm, ABC 0.08 mm, GM 0.06 mm	Automated periodontal ultrasound segmentation and anatomical landmark localization
Anishya et al. [22]	India/2025	Prediction and risk assessment	Neural-network model; Orange Data Mining with ADAM optimizer	Clinical variables from 65 patients undergoing camouflage orthodontic treatment	Prediction of GR risk associated with orthodontic treatment	Overall accuracy 92%; TPR 50%; FPR 50%	Periodontal risk assessment during orthodontic treatment planning
Z et al. [23]	India/2024	Detection	SqueezeNet CNN (feature extraction) + Naive Bayes/Logistic Regression	700 intraoral images: 350 healthy and 350 with GR in the mandibular anterior region	Binary detection of gingival recession	Naive Bayes 96%; logistic regression 100%	Photograph-based screening for gingival recession
Gunpinar et al. [24]	Turkey/2023	Prediction and risk assessment	K-means clustering; Dijkstra’s algorithm	Data from 1057 patients including clinical, demographic, behavioral, and intraoral-scan variables	Periodontal risk stratification and patient-specific 3D simulation of recession-related progression	Quantitative predictive accuracy not reported; specialist satisfaction 36–44%, with 12–20% “very satisfied”	Risk visualization, patient education, and personalized periodontal monitoring
Karagöz et al. [25]	Turkey/2025	Treatment planning and clinical decision support	Logistic regression, multilayer perceptron, and Random Forest	132 teeth with GR; recession and periodontal/preoperative anatomical clinical variables	Selection among four mucogingival surgical approaches	Random Forest overall accuracy 86.4%; highest category-specific accuracy 97.8% for CAF+PRF	AI-assisted selection of a surgical approach for gingival recession
Sun et al. [26]	China/2025	Prediction and risk assessment	YOLO-v8	408 preoperative photographs representing 963 teeth; postoperative photographs used for outcome comparison	Prediction of maximum root coverage following mucogingival surgery	Dice 98.27%; IoU 96.58%; mean error versus 6-month postoperative GM 0.91 ± 1.32 pixels	Preoperative prognosis and patient communication in root-coverage surgery
Chen et al. [27]	Taiwan/2025	Detection and segmentation	DeepLabV3 with ResNet50 backbone	576 intraoral photographs from 32 participants; unstained, reference-retainer, and iodine-stained images	Segmentation of keratinized gingiva and measurement of KGW	Pixel accuracy 93.32%; Dice coefficient 93.30%	Automated assessment and monitoring of keratinized gingiva
Aykol-Sahin et al. [28]	Turkey/2025	Detection and segmentation	ResNet18, ResNet50, and MobileNetV2 with DeepLabV3+	1200 single-tooth images from 50 patients before/after disclosing-agent application	Detection and measurement of keratinized gingiva	Best model (ResNet50) accuracy 91.4%; clinician-model ICC 0.90–0.97	Automated clinical assessment of keratinized gingiva
Liu et al. [29]	China/2026	Detection and segmentation	Vision Transformer (ViT)	1600 patients/1600 max. anterior segments/1600 intraoral photographs	Classification of gingival phenotype (thick, medium, thin)	ViT AUC: 1.00 (Thick), 0.98 (Thin), 0.97 (Medium); F1-scores: 0.92 (Thick), 0.83 (Thin)	Automated gingival phenotype assessment
Kumaralingam et al. [30]	Canada/2025	Measurement and landmark localization	OralSAM (Foundation model)	30 patients/NR teeth/158 IUS videos (~79,000 frames)	Automated measurement of ABL, GT, and ABT on video sequences	ABL ICC 0.893; GT ICC 0.918; ABT ICC 0.848	Automated periodontal ultrasound monitoring
Nguyen et al. [31]	Canada/2021	Measurement and landmark localization	DenseNet201	36 patients/140 teeth/1400 ultrasound images	Localization of CEJ and measurement of CEJ-ABC distance	Mean localization difference 0.26 mm vs. STAPLE; CEJ-ABC correlation R = 0.933	Automated periodontal ultrasound segmentation and anatomical landmark localization

Abbreviations: ABC, alveolar bone crest; AI, artificial intelligence; CAF, coronally advanced flap; CEJ, cementoenamel junction; CNN, convolutional neural network; DSC, Dice similarity coefficient; FPR, false-positive rate; GM, gingival margin; GR, gingival recession; iABL, image-based alveolar bone level; iGH, image-based gingival height; iGR, image-based gingival recession; ICC, intraclass correlation coefficient; IoU, intersection over union; KGW, keratinized gingiva width; ML, machine learning; PRF, platelet-rich fibrin; TPR, true-positive rate. Note: Studies are organized by reference number to facilitate correspondence with the manuscript. Clinical domains reflect the evidence-mapping framework used in the scoping review. For Karagöz et al. [25], 86.4% represents overall model accuracy, whereas 97.8% is the highest category-specific accuracy (CAF+PRF). For Gunpinar et al. [24], clinician ratings describe perceived similarity or realism of generated simulations and should not be interpreted as longitudinal predictive validation. For Sun et al. [26], the postoperative comparison refers to the gingival margin observed at 6 months. For Z et al. [23], the source manuscript reports both a SqueezeNet CNN and performance values for Naive Bayes and logistic regression; these elements are retained without attempting to reconcile them.

**Table 2:** Methodological characteristics and clinical validation of the included studies. Methodological characteristics were mapped descriptively without generating an overall numerical quality score. The table summarizes dataset characteristics, external or cross-device validation, reference-standard and annotation procedures, reporting of class balance, clinical outcome validation, and evaluation within clinical workflows.

Study	Dataset/Setting	Patient-Level Independence/Potential Data Leakage	External/Cross-Device Validation	Reference Standard/Annotation	Class Balance	Clinical Outcome Validation	Clinical Workflow Evaluation
Qi et al. [20]	Periodontal ultrasound images; single-center dataset	NR	Partial/cross-device testing	Expert annotations; reference anatomical landmarks	NA	No	No
Piao et al. [21]	Periodontal ultrasound images; internal dataset plus independent test set	NR	Yes—different ultrasound device and acquisition parameters	Hierarchical annotation: experienced resident with review and refinement by two professors	NA	No	No
Anishya et al. [22]	65 orthodontic patients; structured clinical variables	NR	No	Clinical/database-derived outcome	Potential imbalance; limited reporting	No	No
Mohamed et al. [23]	700 intraoral images; mandibular anterior region	NR	No	Manual expert labeling; inter-rater reliability NR	NR/insufficiently addressed	No	No
Gunpinar et al. [24]	1057 patients; clinical and demographic data plus intraoral scans	NA (Simulation model)	No	Clinical periodontal data; simulations assessed by 25 periodontal specialists	NA	No longitudinal outcome validation; clinician assessment of simulation realism only	No
Karagöz et al. [25]	132 teeth with gingival recession; clinical variables	NR	No	Clinically recorded periodontal and anatomical variables	NR	No	No
Sun et al. [26]	408 photographs representing 963 teeth	NR	No	Single-periodontist annotation; predictions compared with 6-month postoperative gingival margin	NR	Yes—comparison with postoperative outcome at 6 months	No
Chen et al. [27]	576 intraoral photographs from 32 participants; standardized photographic protocols	NR (Split at tooth/image level; potential leakage)	No	Reference retainers and iodine staining used for comparison	NA	No	No
Aykol-Sahin et al. [28]	1200 single-tooth images from 50 patients	NR	No	Clinician-based measurements; agreement with AI assessed	NR	No	No
Liu et al. [29]	1600 photographs	Yes (1 image per patient utilized, ensuring independence)	No	Clinical probe transparency method	Addressed via weighted cross-entropy loss	No	No
Kumaralingam et al. [30]	158 periodontal ultrasound images videos	Yes (Explicitly reported no subject overlap across partitions)	No	Expert manual annotations; inter-rater reliability reported	NA	No	No
Nguyen et al. [31]	1400 periodontal ultrasound images	No (Subject overlap explicitly reported for internal test set)	No	5 clinical raters; probabilistic consensus via STAPLE algorithm	NA	No	No

Abbreviations: AI, artificial intelligence; NA, not applicable; NR, not reported, Note: Methodological characteristics are presented descriptively to map recurring strengths, limitations, and evidence gaps across the included studies. No overall quality score or ranking was assigned. ‘External/Cross-Device Validation’ distinguishes independent or cross-device testing from internal model evaluation. ‘Patient-Level Independence’ indicates whether separation of training, validation, and test data at the participant level was explicitly reported to prevent data leakage. NR indicates that this information was not sufficiently reported to determine patient-level independence. For Gunpinar et al. [24], specialist assessment concerned the perceived similarity or realism of generated recession simulations and did not constitute longitudinal validation of 1–20-year predictions. For Sun et al. [26], clinical outcome validation refers to comparison of model predictions with the postoperative gingival margin at 6 months.

## Data Availability

No new data were created or analyzed in this study. Data sharing is not applicable to this article.

## Supplementary Materials

The following supporting information can be downloaded at https://www.mdpi.com/article/10.3390/jcm15197401/s1. Supplementary Table S1: Complete search strategies and queries used for PubMed/MEDLINE, Scopus, Embase, and Google Scholar. Supplementary Table S2: Selection process for records identified via other methods (backward and forward citation tracking).

## Abbreviations

The following abbreviations are used in this manuscript:

AI	Artificial Intelligence
ML	Machine Learning
DL	Deep Learning
CNN	Convolutional Neural Network
GR	Gingival Recession
KG	Keratinized Gingiva
KGW	Keratinized Gingiva Width
rKGW	Real (clinically measured) Keratinized Gingiva Width
GM	Gingival Margin
CEJ	Cementoenamel Junction
JBI	Joanna Briggs Institute
ABC	Alveolar Bone Crest
iGR	Ultrasonographic Index of Gingival Recession
iGH	Ultrasonographic Index of Gingival Height
iABL	Ultrasonographic Index of Alveolar Bone Level
ABL	Alveolar Bone Level
GT	Gingival Thickness
ABT	Alveolar Bone Thickness
PD	Probing Depth
BOP	Bleeding on Probing
PBI	Papillary Bleeding Index
CAL	Clinical Attachment Level
ICC	Intraclass Correlation Coefficient
DSC	Dice Similarity Coefficient
IoU	Intersection over Union
TPR	True Positive Rate
FPR	False Positive Rate
NA	Not Applicable
NR	Not Reported
CAF	Coronally Advanced Flap
CTG	Connective Tissue Graft
PRF	Platelet-Rich Fibrin
CAF+PRF	Coronally Advanced Flap with Platelet-Rich Fibrin
GUG	Gingival Unit Graft
XAI	Explainable Artificial Intelligence
