# Supplementary material for: Artificial Intelligence in Gingival Recession Assessment and Treatment Planning: A Scoping Review
Source: J Clin Med. 2026 Sep 23;15(19):7401. doi: 10.3390/jcm15197401 (PMC13666376; doi:10.3390/jcm15197401)
Supplement: Supplementary file 1 [file jcm-15-07401-s001.zip › Supplementary Table S1.pdf]

Table S1. Complete search strategies and queries used for PubMed/MEDLINE, Scopus, Embase, and Google Scholar.

Database	Search ID	Search Query (String with Boolean operators and tags)	Hits / Results
**PubMed / MEDLINE**	#1	("Artificial Intelligence"[Mesh]) OR "Deep Learning"[Mesh]) OR "Machine Learning"[Mesh]) OR "Neural Networks, Computer"[Mesh] OR ("artificial intelligence"[tiab] OR "machine learning"[tiab] OR "deep learning"[tiab] OR "neural network*"[tiab] OR CNN[tiab])	**576,636**
	#2	("Gingival Recession"[Mesh] OR ("gingival recession"[tiab] OR "gingival recessions"[tiab] OR "gingival atrophy"[tiab] OR "gingival atrophies"[tiab] OR "atrophy of gingiva"[tiab] OR "gingiva atrophies"[tiab])	**6,294**
	#3	((((((("Classification"[Mesh]) OR "Clinical Protocols"[Mesh]) OR "Diagnosis"[Mesh]) OR "Diagnostic Imaging"[Mesh]) OR "Forecasting"[Mesh]) OR "Prognosis"[Mesh]) OR "Radiography"[Mesh] OR "Therapeutics"[Mesh]) OR (diagnos*[tiab] OR classif*[tiab] OR segmentation[tiab] OR prognos*[tiab] OR "treatment planning"[tiab]) OR Radiogra*[tiab])	**16,556,543**
	#4	#1 AND #2 AND #3	**10**
**Scopus**	#1	(TITLE-ABS-KEY("artificial intelligence" OR "machine learning" OR "deep learning" OR "neural network" OR "computer neural network" OR "convolutional neural network*" OR CNN)) AND (TITLE-ABS-KEY("gingival recession" OR "gingival recessions" OR "gingival atrophy" OR "gingival atrophies" OR "atrophy of gingiva" OR "gingiva atrophies")) AND (TITLE-ABS-KEY(diagnos* OR classif* OR segmentation OR prognos* OR "treatment planning" OR radiogra* OR "diagnostic imaging" OR "radiographic imaging" OR "clinical protocol*" OR therapeutic* OR forecast*)) AND (SUBJAREA(DENT) OR SUBJAREA(MEDI))**	**15**
**Embase**	#1	'artificial intelligence'/exp OR 'deep learning'/exp OR 'machine learning'/exp OR 'neural network'/exp OR 'artificial intelligence':ti,ab,kw OR cnn:ti,ab,kw OR 'computer neural network*':ti,ab,kw OR 'convolutional neural network*':ti,ab,kw OR 'deep learning':ti,ab,kw OR 'machine learning':ti,ab,kw OR 'neural network*':ti,ab,kw	**1,006,542**
	#2	'gingiva recession'/exp OR 'atrophy of gingiva':ti,ab,kw OR 'gingiva atrophies':ti,ab,kw OR 'gingival atrophies':ti,ab,kw OR 'gingival atrophy':ti,ab,kw OR 'gingival recession':ti,ab,kw OR 'gingival recessions':ti,ab,kw OR 'mucogingival defect':ti,ab,kw OR 'mucogingival defects':ti,ab,kw OR 'recession defect':ti,ab,kw OR 'recession defects':ti,ab,kw	**5565**
	#3	'classification'/exp OR 'clinical protocol'/exp OR 'diagnosis'/exp OR 'diagnostic imaging'/exp OR 'forecasting'/exp OR 'prognosis'/exp OR 'radiography'/exp OR 'therapy'/exp OR classif*:ti,ab,kw OR 'clinical protocol*':ti,ab,kw OR diagnos*:ti,ab,kw OR forecast*:ti,ab,kw OR prognos*:ti,ab,kw OR radiogra*:ti,ab,kw OR segmentation:ti,ab,kw OR therapeutic*:ti,ab,kw OR 'treatment planning':ti,ab,kw	**24,591,311**
	4#	#1 AND #2 AND #3	**18**
**Google Scholar**	#1	("artificial intelligence" OR "machine learning" OR "deep learning" OR "neural network" OR "neural networks" OR CNN) AND ("gingival recession" OR "gingival recessions" OR "gingival atrophy" OR "atrophy of gingiva" OR "mucogingival defect" OR "mucogingival	**2860**<br>First **200** entries sorted by relevance were examined for eligibility, of which **47**

Database	Search ID	Search Query (String with Boolean operators and tags)	Hits / Results
		defects" OR "recession defect" OR "recession defects") AND (diagnosis OR diagnostic OR diagnosing OR classification OR classifying OR "clinical protocol" OR "clinical protocols" OR prognosis OR prognostic OR "treatment planning" OR segmentation OR "diagnostic imaging" OR radiograph OR radiography OR radiographic)	were retained for inclusion in the initial record set.
