# Supplementary material for: Artificial Intelligence in Gingival Recession Assessment and Treatment Planning: A Scoping Review
Source: J Clin Med. 2026 Sep 23;15(19):7401. doi: 10.3390/jcm15197401 (PMC13666376; doi:10.3390/jcm15197401)
Supplement: Supplementary file 1 [file jcm-15-07401-s001.zip › Supplementary Table S2.pdf]

Table S2. Selection process for records identified via other methods (backward and forward citation tracking).

Seed study	Direction	Candidate article (Author, Year, Title)	DOI	Duplicate?	Title/abstract eligible?	Full text eligible?	Reason for exclusion
**Chen et al., 2025**	Backward	Aykol-Sahin, G., O. Yucel, N. Eraydin, G. C. Keles, U. Unlu, and U. Baser. 2024. "Efficiency of Oral Keratinized Gingiva Detection and Measurement Based on Convolutional Neural Network." Journal of Periodontology.	https://doi.org/10.1002/ JPER.24-0151.	Yes			Duplicate
**Chen et al., 2025**	Backward	Li, G.-H., T.-C. Hsung, W.-K. Ling, W. Y.-H. Lam, G. Pelekos, and C. McGrath. 2021. "Automatic Site-Specific Multiple Level Gum Disease Detection Based on Deep Neural Network."	https://doi.org/10.1109/ ISMICT51748.2021.9434936.	No	Yes	No	Outcome not related to gingival recession
**Qi wet al., 2025**	Backward	Nguyen K-CT, Le BM, Li M, et al. Localization of cementoenamel junction in intraoral ultrasonographs with machine learning. J Dent. 2021;112:103752.	https://doi.org/10.1016/j.jdent.2021.103752	No	Yes	Yes	
**Piao et al., 2025**	Backward	K.T. Nguyen, B.M. Le, M. Li, F.T. Almeida, P.W. Major, N.R. Kaipatur, E.H.M. Lou, K. Punithakumar, L.H. Le, Localization of cementoenamel junction in intraoral ultrasonographs with machine learning, J. Dent. 112 (2021) 103752,	https://doi.org/ 10.1259/dmfr.20210363	Yes			Duplicate
**Qi et al., 2025**	Forward	L.Kumaralingam, 2026, Automatic Assessment of PeriodontiumComplex in Intraoral Ultrasound Videos	https://doi.org/10.1177/00220345251387713 Journal	No	Yes	Yes	
**Qi et al., 2025**	Forward	L.Kumaralingam, 2026, OralSAM: One-Shot Segmentation for Intraoral Ultrasound Videos with Adaptive Feature Correlation and Self-prompting Strategy	DOI: 10.1007/978-3-032-04981-0_37	No	Yes	No	Description of model development, no clinical applicability
**Aykol-Sahin et al. 2025**	Forward	Chen et al.,2025, AI -Driven Detection and Measurement of Keratinized Gingiva in Dental Photographs: Validation Using Reference Retainers.	https://doi:10.1111/jcpe.14164.	Yes			Duplicate
**Aykol-Sahin et al. 2025**	Forward	Yurdakurban et al, Automated periodontal assessment in orthodontic patients: a dual CNN framework	DOI: 10.1007/s00784-025-06410-5	No	Yes	No	Outcome not related to gingival recession
**Aykol-Sahin et al. 2025**	Forward	Karagollu et al. Optimized AI Models for Gingiva Analysis and Real-Time Dental Segmentation on Mobile Devices	DOI: 10.1007/978-3-030-60334-2_23	No	Yes	No	Outcome doesn't include detection, measurement, prediction or treatment planning of GR
**Aykol-Sahin et al. 2025**	Forward	Liu et al. Automated assessment of gingival biotype using deep learning on intraoral photographs	https://doi:10.7717/peerj-cs.3518.	No	Yes	Yes	

**Aykol-Sahin et al. 2025**	Forward	E Yurdakurban, 2025, Automated periodontal assessment in orthodontic patients: a dual CNN framework	https://doi.org/10.1007/s00784-025-06410-5	No	No		Outcome not related to gingival recession
